# Adolescent Assent and Reconsent for Biobanking: Recent Developments and Emerging Ethical Issues

**DOI:** 10.3389/fmed.2021.686264

**Published:** 2021-07-09

**Authors:** T. J. Kasperbauer, Colin Halverson

**Affiliations:** Indiana University Center for Bioethics, Indiana University School of Medicine, Indianapolis, IN, United States

**Keywords:** informed consent, assent, biobanking, autonomy, learning health systems

## Abstract

Research biobanks that enroll minors face important practical, ethical, and regulatory challenges in reconsenting participants when they reach the age of 18. Federal regulations governing research in the United States provide minimal guidance and allow for a range of practices, including waiving the requirement to obtain reconsent. Some commentators have argued that institutional review boards should indeed grant such waivers, given the low risks of biobank-based research and the impracticality of contacting all participants when they turn 18. There is also significant ethical debate about the age at which adolescents can make authentic, autonomous decisions regarding their research participation. This paper reviews these issues in detail, describes the current state of the ethical discussion, and outlines evidence-based policies for enrolling minors into research biobanks.

## Introduction

In the United States and many other countries, individuals under the age of 18 are not legally allowed to provide full consent to participate in research. Instead, parents or guardians must provide that initial permission. Once the participants reach the legal age of majority, however, their legal status abruptly changes; they are now able to consent for themselves (1, 2). In longitudinal studies or in repositories that store biospecimens over years or decades, a key issue is thus what to do when participants enrolled as minors reach the age of majority. Is reconsent necessary, and if so, how should it be obtained? Federal laws governing research in the United States allow for a range of practices concerning reconsent. Ethically, no consensus has yet been reached on the topic, with increasing debate over the age at which adolescents can make authentic, autonomous decisions about their research participation. Research biobanks thus face important practical, ethical, and regulatory challenges in responding to this issue, which we explore in this paper.

While biobanks may reconsent adolescent participants as a matter of policy, an increasingly popular option in the U.S. is to request that Institutional Review Boards (IRBs) waive the requirement to reconsent under certain conditions. Some commentators have argued in favor of IRBs granting such waivers, given the low risks of biobank-based research and the impracticality of contacting all participants when they turn 18 (3, 4). Nonetheless, the participation of minors in such research raises a number of ethical issues. For instance, biospecimens are often collected for long-term, currently unspecified usage. A waiver of consent means that biobanks can indefinitely retain biospecimens from minors without any further engagement beyond the initial permission of their parent or legal guardian. Moreover, a number of health systems have begun collecting pediatric samples during the course of patient care, linking specimens to sensitive clinical data contained in patients' electronic health records (5, 6). Waiving consent entails, in principle, that all of this information can be used in a wide range of medical research based on a single interaction with researchers, and potentially when the participants are too young to understand the decision their guardians are making on their behalf. Finally, other possible uses of biospecimens and associated information include genetic sequencing, commercialization, and linking to third-party datasets as part of secondary research. These possible uses place heavy demands on the assent process and raise important questions about how to reconsent participants responsibly, including how to determine whether a consent waiver is appropriate.

Finding solutions to these challenges is particularly pressing given the trajectory of growth in pediatric biobanking and the importance of adolescent participation specifically for treating pediatric diseases. An often-cited study from 2013 found that more than 200 biobanks in the U.S. store biospecimens from minors (7). Given the rise of pediatric biobanking over the last decade, thousands of pediatric participants across the country may require reconsent in the next few years^1^. There is significant research potential in their samples, but there is also an important ethical and practical risk, if researchers were to violate the trust of adolescent patients and research participants. Despite an absence of clear regulatory guidance on reconsenting adolescent biobank participants, a range of new strategies has been developed to address the issues outlined above. This paper reviews these issues in detail, describes the current state of the ethical discussion, and outlines evidence-based policies for enrolling minors into research biobanks.

## Historical and Regulatory Context

The ethical foundation of informed consent is the principle of autonomy, that is, the right for people to control their own lives and make decisions for themselves (9). Accordingly, debates about including minors in medical research have historically focused on their capacity to make autonomous decisions. Current discussions of assent and reconsent for minors in research can be traced back to different perspectives on autonomy within the Nuremberg Code (1948), the Declaration of Helsinki (1964), and the Belmont Report (1979).

Due to the atrocities of Nazi medicine, the Nuremberg Code opposed medical research on children, among other vulnerable populations. Because children were viewed as incapable of providing voluntary consent, they could not participate in medical research. In contrast, both the Declaration of Helsinki and the Belmont Report created space for the possibility of pediatric assent. The Belmont Report in particular expounded on the importance of respecting the dissent of children and adolescents, even if they lack the capacity to provide full-fledged consent (10). On the subject of informed consent, the Belmont Report states that “respect requires giving [infants and young children] the opportunity to choose to the extent they are able.” In addition to requiring permission from a responsible third party, the Report protected minors by treating their objections to research participation as authoritative (albeit with some qualifications, such as when research offered the only option for treatment). The Belmont Report became the basis for the creation of the United States' Federal Policy for the Protection of Human Subjects (known as the Common Rule), which along with other federal regulations (45 CFR 46) formally protect minors' right to assent and dissent. Major international guidelines on research ethics now also recommend obtaining affirmative assent from minors before including them in medical research (11, 12).

The question of autonomy has continued to play a central role in recent discussions about changing the Common Rule. Concerns about autonomy and respect for participants led to proposals, in the 2015 Notice of Proposed Rule Making, that would require (1) reconsent for secondary usage of specimens and associated information, and (2) consent for the use of de-identified specimens and genetic information (13). If these proposals had been accepted, they would have had significant implications for pediatric biobanking. Reconsenting minors would have become mandatory, regardless of whether the specimens had been de-identified, and failure to reconsent would require those specimens be destroyed. Biobanks often contain samples that were collected a decade or more earlier and may not maintain any sustained contact with participants. As a result, they often lack accurate, up-to-date contact information, which would have made this proposed requirement a significant burden on research. It is perhaps unsurprising, then, that these proposals were excluded from the final version, largely due to concerns that the practical challenges of reconsenting such a large number of participants would have had a significant, negative impact on medical research and a chilling effect on the progress of science (14).

The final version of the Common Rule, which went into effect in 2019, does not specifically address biobank reconsent. Reconsent for minors is encompassed by the requirement that legally effective consent can only be obtained from adults. However, this requirement can be circumvented by obtaining a consent waiver from the relevant IRB. A researcher requesting such a waiver must demonstrate that the following conditions apply to their proposal (45 CFR 46.116):

- the research involves no more than minimal risk to the subjects,
- the research could not practicably be carried out without the requested waiver or alteration,
- if the research involves using identifiable private information or identifiable biospecimens, the research could not practicably be carried out without using such information or biospecimens in an identifiable format,
- the waiver or alteration will not adversely affect the rights and welfare of the subjects, and
- whenever appropriate, the subjects or legally authorized representatives will be provided with additional pertinent information after participation.

Determining whether a research study does in fact meet these conditions is entirely the decision of the governing IRB^2^. Additional guidance from the Office of Human Research Protections recommends reconsent at the age of majority but also supports the use of consent waivers^3^. This means, in principle, that biobanks can seek to waive reconsent for all adolescent participants when they reach the age of majority. In commenting on the issue of reconsent both before and after the revisions to the Common Rule, Kyle Brothers and colleagues have argued that there should be a presumption in favor of pursuing reconsent but that there is no affirmative duty to do so [i.e., reconsent does not amount to a moral obligation (3, 15, 16)]. They reason that allowing consent waivers while still making an attempt to reconsent promotes both adolescents' autonomy and progress in medical research.

It is unclear how these professional and expert attitudes toward consent align with those of actual pediatric research participants. Our understanding of the experiences and perspectives of these participants and their guardians has historically been limited. In the context of biobanks, this is because there has been scant empirical work—until recently—on the attitudes of adolescents toward contributing to biobanks and what such participants might prefer regarding assent and reconsent. While consent waivers might seem to threaten adolescents' autonomy, an emerging trend is to use adolescents' preferences to inform biobank policies, including whether a consent waiver is appropriate. Criteria 2 and 3 of the new Common Rule's consent waiver policy (above) refer to practicability, and one major practical challenge is recontacting participants once they reach the age of majority. By soliciting adolescents' opinions on recontact when they reach 18, biobanks can better position themselves to evaluate whether consent waivers are appropriate. In the next section, we summarize existing empirical work on adolescent preferences that can inform biobank consent policies. Such empirical work is necessary to weigh the costs and benefits of consent options in order both to advance medical research and to honor adolescent participants' autonomy.

## Patient and Participant Preferences

In general, studies have shown that adolescents are interested in contributing to biobanks, want to exert control over their specimens and associated information, and feel confident in their decision-making abilities (17–19). However, these attitudes have a variety of implications for reconsent preferences. Over a decade ago, Goldenberg et al. (20) surveyed 1,186 adult patients in the U.S. about their thoughts on the collection of pediatric samples. A slight majority (54%) was explicitly opposed to reconsent and only 26% objected to continued use of samples in cases where recontact was unsuccessful (e.g., if contact information was outdated). Recent studies have found greater support for reconsent but still show a pattern consistent with Goldenberg et al.'s results: Adolescents express a slight preference for reconsent but also simultaneously support retaining specimens, should recontact prove unsuccessful or especially burdensome.

For example, among 211 adolescents in British Columbia, 55% said that reconsent at the age of majority was either “important” or “very important” (21). A greater percentage (67%) of their parents thought the same. However, when asked whether biospecimens should continue to be used if attempts to obtain such consent were unsuccessful, 54% of adolescents compared to 47% of their parents said yes, that keeping the specimens was acceptable.

Murad et al. (22) recently found similar results in the U.S. context. The majority of the 15- to 17-year-olds they interviewed supported reconsent for biobanking; only half, however, thought that specimens should be discarded if recontact was unsuccessful. Interestingly, these interviewees viewed reconsent as particularly important for children who assented between the ages of eight and 12. In addition to perceiving adult consent to be a fundamental right, they thought it was necessary to acknowledge that one's comprehension of the implications of biobank participation increases with age, that younger children may have disagreed with their parents' decision, and that younger children may have forgotten that they had been enrolled.

Adolescents with diagnosed medical conditions or rare diseases seem especially supportive of reconsent. Individuals with rare diseases are often interested in biobanking because they believe it holds the potential for finding new treatments for their condition. However, with this increased support come expectations regarding proper treatment of their specimens (23). For example, a study of adolescents with rare diseases in 16 different countries found that the majority supported reconsent (24). Not seeking their consent, participants explained, violated their autonomy as well as the agreement they thought they had entered with the biobank. Failing to reconsent was viewed as equivalent to breaking the contract created by their initial assent. Similarly, a survey of adults in Australia who had donated to a cancer biobank as minors found that 18 of the 30 participants supported the idea of reconsent (25). Even though most had forgotten that they had even contributed to the biobank, they still wanted to be approached again to give their legal consent.

Adolescents' attitudes toward consent waivers for biobanking have not been investigated empirically. However, the research summarized above suggests that adolescents may indeed view consent waivers as illegitimate, at least without a good-faith attempt to recontact them before pursuing an alternative to explicit consent. Waiving consent for every biobank participant as an official policy risks destroying trust in biobanks. Of course, like any biobank participant, adolescents may forget about their specimens over time, as has been demonstrated with adult participants (25). However, should they later realize that the biobank retained their samples without pursuing reconsent, they may consequently decide to withdraw or refuse to participate in further research. This has the potential for broader-reaching effects, should these participants communicate their distrust to other members of their communities or social networks. The empirical evidence reviewed here suggests that this possible outcome could be avoided by further engagement when participants reach the age of majority.

One salient issue in life stage-based research is whether apparent differences are due to particular developmental stages or to a generational shift in attitudes. Some of the findings in the literature reviewed above seem to have demonstrated differences between adult, late adolescent, and early adolescent perspectives. However, it is unclear to what to attribute these differences, as it has both been suggested that younger generations (e.g., Gen Z) have different perspectives on ethical issues like privacy (26) and that people at earlier stages of life—regardless of generation—have different interests and experience different ethical obligations than do their seniors (27).

## Developing Capacity to Make Consent Decisions

Analyses of the ethics of assent and reconsent have focused on adolescents' developing capacity to make decisions for themselves, especially regarding their bodies and personal information (16, 28–31). Providing opportunities to make independent decisions in the context of medical research can perhaps help facilitate the development of these capacities. However, the challenge comes in determining the types of decisions appropriate to different stages of development. Singleton and colleagues explain, “Along the developmental trajectory, children move steadily from minimal autonomy to robust autonomy; respect for the developing autonomy of children is future-focused” (32). The assent process is meant to accommodate minors' growing decision-making capacity by reserving major or particularly demanding decisions until they are more cognitively capable. Presenting a difficult choice too early can lead to poor decisions that adversely affect the future options available to an individual. For example, asking young children to assent to a biobank that plans to waive reconsent might be seen as posing this sort of risk. Consequently, adult-like consent might facilitate autonomy for some adolescents while undermining it for others, based on how elaborated or refined their capacities are at a given stage in their lives.

In certain states and under certain rare circumstances, pediatric participants can be labeled “mature minors,” legally allowed to consent to low-risk procedures like biobank participation, as long as they have “sufficient intelligence to understand and appreciate the consequences” of those decisions (33). Some studies have indicated that the cognitive ability to make medical decisions at the same level as adults emerges around age 14, and potentially as young as 12 (34, 35). For example, using the MacArthur Competency Assessment Tool for Clinical Research (MacCAT-CR), a standard capacity assessment tool, McGregor and Ott (36) found that even young adolescents were able to process biobank consent information at a level equivalent to that of adults. The MacCAT-CR consists of four subscales that measure participant understanding, appreciation of the consent situation, ability to reason about consent information, and ability to express a choice. Combining scores from these four subscales provides a measure of overall capacity to consent. After controlling for health literacy and affluence, McGregor and Ott found no significant differences in overall capacity between individuals aged 12–24.

An implication of this research is that biobank assent materials given to teenagers can be written in a fashion similar to those given to adults for consent (15)^4^. Individual variation in capacity must also be taken into account—some adolescents will lack capacity, as will some adults—but in general this evidence suggests that many adolescents can meet the same minimal capacity threshold as young adults. Children younger than 12, meanwhile, should receive different materials [with 5 years of age as potentially the low end of the range for meaningful assent (38–40)]. A further implication is that reconsent for biobanks may be unnecessary if participants are enrolled after this threshold. McGregor and Ott conclude, based on their study described in the previous paragraph, that reconsent is indeed unnecessary because adolescents demonstrate “adequate understanding of biobanking information, appreciation for how biobanking would affect them personally, and ability to apply reasoning skills such as weighing risks and benefits and creating a logical argument for their choice” (p. 19). If a teenager is able to make a meaningful decision to participate in a biobank, then waiving consent at the age of majority may not deprive them of autonomy, because their previous assent was authentic and would be unlikely to change over the course of a longitudinal study.

However, this conclusion is complicated by the fact that these participants do not enroll on their own; they only do so with the permission of their parents or guardians, who can strongly influence assent decisions. Studies of assent and consent processes for genetics research have found that guardians tend to dominate the conversation, with adolescents mostly asking trivial, practical questions (31, 41, 42). Consequently, adolescents' preferences have less weight in the final decision. Reconsent may therefore be seen as especially important because adolescents' decision-making capacity is often indistinguishable from that of legal adults, yet they may have only minimal influence on their guardians' decisions. Reconsent could provide another opportunity for adolescents to control their information, in case the decision to participate was made with only their limited input.

There are other relevant developmental factors to consider beyond the basic cognitive capacity to understand consent materials and the consent situation. The adolescent brain continues to develop and mature into young adulthood. While adolescents may have a similar level of cognitive capacity to that of young adults, they do not always use those abilities optimally (43, 44). Consequently, adolescents' abilities may still improve appreciably throughout early adulthood as other elements of decision-making continue to develop. Moral values and a sense of moral identity are also in flux during the teenage years, such that adolescents' attitudes toward biobanks may change significantly between the time of assent and the age of majority (45, 46). Even if children understand the biobank information presented to them during assent, their interpretation of that information at the age of 18 may be very different. In medical ethics, a minimum level of consistency and stability in a participant's values is a cornerstone in judging the appropriateness of decision-making, especially for decisions concerning minors (2).

Changing capacities and moral values may also suggest that re-*assent* may be necessary for minors, and perhaps even reconsent throughout adulthood, beyond just the age of 18. Berkman et al. (28) argue that if facilitating autonomy is the justification for reconsent, then it may be appropriate to present consent information at multiple stages during the long course of an individual's participation in biobank-based research (They acknowledge that this would impose significant burdens on research, suggesting that more is at stake ethically than merely preserving the autonomy of the participants.). It is also well-established that both adults and adolescents misunderstand significant details about biobank participation [e.g., that specimens are stored for indefinite, long-term uses and will be shared outside the biobank (22, 47, 48)]. We must thus acknowledge that even the most mature participants still need assistance to understand the implications of biobank-based research. Consistent with relational views of autonomy, obtaining consent for research is not just about respecting individuals' choices but also supporting and empowering individuals based on their beliefs and needs (49). The involvement of conscientious third parties can actually help to promote the participant's autonomy in such decisions. Reconsent at 18 is only one checkpoint to provide this assistance; changing capacities throughout the human lifespan could justify additional engagement from biobanks. Below we discuss ways that some biobanks are beginning to address these issues.

## Assent and Reconsent Strategies

There are two basic routes to enroll minors into a biobank through the assent/consent process: (1) assent followed by reconsent at the age of 18, or (2) assent combined with a consent waiver. In either case, adolescents—like adults—can withdraw from participation at any time, before or after turning 18.

Biobanks can pursue and communicate these options to participants in different ways. One possible strategy is to place conditions on consent waivers. For example, Hartsock et al. (4) argue that consent waivers are appropriate for participants older than 12 if a good faith attempt has been made to recontact them (They suggest three attempts would be sufficient; children enrolled before 12 years of age would still require successful reconsent to remain in the biobank after reaching legal majority). These conditions on waivers would essentially require biobanks to demonstrate that recontact is indeed impracticable. It is unclear, however, what should count as “impracticable,” and relevant guidance on the issue is lacking (3).

Biobanks can also communicate to participants that their consent was waived because they met certain conditions, such as being over a certain age at the time of enrollment. The consent waiver can essentially operate like an opt-out consent process, where participants are informed at the age of majority that their specimens will be kept unless they explicitly choose to have their samples removed [e.g., by submitting a request detailing this choice as opposed to taking no action (29, 30)]. Participants could also be notified that failure to respond to three attempts at recontact (Hartsock and colleagues' “good faith effort”) will result in an automatic consent waiver, meaning their specimens will be retained unless they explicitly withdraw at some future time. Biobank information can also be made easily available online, in case participants later wish to change their decision or simply learn more about the research being conducted using their sample (50).

Another strategy is to ask adolescents what they prefer regarding reconsent. Although there is no regulatory mechanism in the U.S. that allows adolescents to waive reconsent unilaterally, researchers and IRBs can still take their preferences into account when determining whether a waiver is appropriate. As discussed above, adolescents can make meaningful decisions regarding their participation. For example, consent forms could include options to waive consent entirely, waive consent after three attempts at recontact, or discard samples if recontact is unsuccessful. These options could furthermore be tailored according to age, with older adolescents receiving progressively more control over future decisions.

The regulatory uncertainties related to reconsent have led to a wide array of strategies in managing minor biobank participants as they reach the age of legal majority. However, as biobanks begin to offer the option of returning the results of genetic tests to participants, added pressure has been placed on the issues of assent and reconsent. As a result, many pediatric biobanks have developed robust and explicit policies regarding reconsent. In the following, we consider some of the reconsent practices that are currently being implemented by a variety of research institutions.

The eMERGE-III study was developed specifically to return genetic test results to patients at a number of institutions across the United States. Though most participants in the study were adults at the time of enrollment, member sites such as the Children's Hospital of Philadelphia and Cincinnati's Children's Hospital and Medical Center each aimed to recruit 3,000 minors, with the goal of treating childhood-onset genetic diseases (51). A study of the eMERGE sites that collected pediatric biospecimens found that all sites had attempted to recontact participants when they turned 18 (38). If recontact was unsuccessful, the patient's information was de-identified but the biospecimen was retained. It therefore remained possible for these sites to conduct research on such a patient's sample, but de-identification prevented any return of results or direct link to the individual. However, one eMERGE site asked 13- to 17-year-olds to choose what they would prefer if the biobank's attempts at recontact were unsuccessful. During assent, they could indicate with the check of a box whether they wanted the samples retained or destroyed. Participants younger than 13 did not receive any such choice. Failure to recontact a particular participant automatically meant that his or her sample would be discarded.

Geisinger's MyCode program similarly collects specimens with the intent of genetic sequencing to improve patient outcomes (52, 53). As of March 2020, the program had consented over 5,000 pediatric patients for biobanking and genetic analysis, a small number of whom had a pathogenic variant that was considered actionable (54). However, none of the parents had consented for that information to be placed into their clinical record. Adolescents aged 15–17 who enroll in the program receive a different assent form from that given to other minors. The adapted assent allows them to select responses separately from their parents, such that older adolescents can decide to enroll in the program independent of their parents.

To improve recruitment and recontact, a number of institutions have incorporated biobank consent materials into electronic health records (EHRs) and health portals. Combining clinical and research information in this way facilities the creation of learning health systems, where patients contribute to research and, in turn, the results of that research improve patient care (55). Prior to a doctor's appointment, Boston Children's Hospital prompts every patient (or their guardians) via email with the option to enroll in the Precision Link Biobank. The patient's response is then logged in their patient portal (5, 56). Pediatric participants may also be recruited to a separate program that returns genetic results to patients (57). All such patients are reconsented to the biobank when they reach the age of 18. If contact is unsuccessful, the biobank allows no additional research using those patients' samples and associated data, beyond what has already been shared with researchers.

BioVU at Vanderbilt is unique in that it began as an opt-out biobank for both minors and adults (58). BioVU is now opt-in, requiring prospective consent for enrollment of both minors and adults. As of 2012, it contained over 16,000 pediatric samples^5^. They decided that their policy would be to retain samples indefinitely unless the pediatric guardian or the participant, when he or she turns 18, should choose to withdraw.

## Technological Tools

A variety of technological advancements are currently transforming biobank consent. E-consent and other digital tools are rapidly replacing paper consent in order to increase engagement with consent materials (59–61). Paper is traditionally the most frequently used method, and manages to fulfill all the basic requirements of consent; however, these new digital tools have the potential to ease and even improve the consent process. They can serve numerous functions for refining assent and reconsent, including tracking for recontact purposes, presenting additional choices to adolescents, informing participants about ongoing research, and evaluating comprehension of consent materials.

An emerging trend in pediatric biobank consent is to use videos and other multimedia formats to enhance consent information. While videos cannot resolve reconsent challenges on their own, they can help to clarify assent and reconsent procedures for participants in a more engaging way than paper consent. A number of biobanks have produced professional videos, including animations, to explain the basics of biobank participation (62). Videos can incorporate consent information itself, as Le Bonheur Children's Hospital in Memphis^6^ and Nicklaus Children's Hospital in South Florida^7^ have done. They can also be used to supplement consent information or illustrate biobank procedures. For example, the video used by Boston Children's Hospital is included alongside their online consent as an introduction to the biobank^8^. The University of Michigan's pediatric biobank created an illustrated version of their assent and consent forms in order to better convey information about the biobank to an adolescent audience (63). They also created an “augmented-reality,” gamified version of their assent materials that tells the story of a child learning about health research.^9^ Pediatric patients can choose different “adventures” in the story that teach them about various elements of biobank participation.

The effectiveness of video consent for adolescent enrollment in biobanks is understudied. Research with adults indicates that interactive video-based consent for biobanking can improve comprehension, compared to standard paper consent (64). A recent study of parents enrolling their children into Michigan's blood spot biobank (BioTrust) found that both video and app-based consent produced higher rates of comprehension, compared to standard text-based consent (65). These studies suggest that video consent may also improve minors' uptake of consent information, though this currently remains an untested hypothesis.

As discussed above, one of the main challenges for reconsenting adolescents is maintaining accurate contact information. Reconsent requires a system for tracking these participants until they reach 18, and potentially tracking them across different health systems as families move or choose new providers. Some commentators have argued that if reconsent is to be mandatory, then the NIH and other agencies must also enable the implementation of such systems [e.g., through dedicated funding (28)].

The solution to this problem of recontact that some health systems have pursued, as mentioned above, is to link participant information to their EHRs. Such links reduce some of the burden of tracking participants and maintaining valid contact information. However, linking to EHRs also raises privacy concerns, given the direct, long-term connection that would exist between genetic data and other sensitive information contained in EHRs. Such a link may also exacerbate existing concerns about confidentiality in adolescent EHRs for biobanks that return genetic results to patients (66, 67). There are also options to create portals outside of EHRs that can provide additional features beyond facilitating recontact. For example, dynamic consent portals can be used to log participant choices and potentially allow those choices to be modified over time (3, 68). Biobanks can use public-facing websites that automatically inform participants whenever their samples are used and allow them to opt out of specific uses (69). For instance, the dynamic consent portal that has been pilot tested with BioTrust, the Michigan blood spot biobank mentioned above, allows ongoing control of children's specimens (70). Though it has not actually been implemented, the consent portal would allow guardians to opt out of research, commercialization, and other downstream uses of the child's specimens. There are also general registry platforms, such as the PEER program (Promise for Engaging Everyone Responsibly)^10^, that a biobank could tailor for their participants.

However, dynamic consent portals have been surprisingly slow to reach the implementation stage. A variety of logistical and practical challenges appears to have made biobanks reluctant to develop these portals. For example, some studies of dynamic consent portals indicate that adults anticipate changing their communication preferences over time, which could hinder longitudinal research with their data (71, 72). Allowing adolescents to change their preferences throughout their teenage years or after they reach 18 would pose a similar problem. Nonetheless, dynamic consent portals could provide a robust system for tracking and communicating with adolescents over the course of their participation, potentially supporting participants' desire for recontact.

As we have discussed, adolescent assent and reconsent raise a host of challenges for biobanks. While empirical work in this area is still nascent, certain evidence-based options are clear. Table 1 summarizes key points that biobanks may consider following in order to address these challenges and to develop ethical and responsible policies.

**Table 1:** Key considerations for adolescent assent and reconsent.

**Points to consider**
1. Adolescents can make meaningful, authentic decisions concerning their participation in biobank-based research.
2. Adolescents seem to prefer being approached for reconsent.
3. Adolescents also seem to support the biobank's retention of specimens if recontact is unsuccessful.
4. Placing conditions on waivers, such as good faith attempts at recontact, can preserve autonomy within the waiver process without significantly burdening research.
5. Asking adolescent participants their preference regarding reconsent waivers can help inform relevant policy decisions.
6. Reconsent policies should take into account adolescents' cognitive capacity and developing moral values.
7. Reconsent policies should also consider the potential controlling influences of parents or legal guardians in pediatric assent.
8. Videos and interactive consent programs can improve engagement and education about biobank participation.
9. Linking biospecimens to patient EHRs can facilitate recontact when participants reach 18 years of age, though there are also privacy concerns to consider.
10. Dynamic consent and related portals may be able to help communicate with and solicit consent decisions from participants, in addition to facilitating recontact when participants reach 18 years of age.

## Discussion: Areas for Further Study

Continued research is needed in order to improve the experiences of pediatric participants when enrolling in biobanks. Studies that focus on the issues affecting this specific population are necessary to support this important and growing area of biobank-based research. We see four broad domains where additional investigation may prove particularly important in the coming years.

First, a better understanding of how adolescent preferences contrast with those of adult participants would be highly informative for a more developmentally-sensitive ethics of pediatric assent. To what degree do adolescents' preferences regarding biobank participation change over time? How do the specific social conditions of childhood and adolescence affect relevant values? What are the unique determinants of trust for adolescents enrolling in biobanks and other health data repositories? What are their preferences regarding consent waivers and the acceptable conditions under which such waivers can be sought?

Second, communication with adolescents about medical research may require special features and accommodations. What style and frequency of ongoing communication about biobank research is ideal, both before and after the participant reaches the age of 18? How can researchers best communicate the complexities of commercialization and downstream uses of biospecimens, including sharing genomic data from biobanks? How do current recommendations for specific language (such as the NIH's 2015 Genomic Data Sharing policy) correspond to the unique needs of an adolescent audience? Many institutions already struggle with returning complex results, such as variants of unknown significance and reclassified results. How might such information be communicated to younger participants? And when should such a discussion take place: at initial assent or at the time of reconsent, when the participant has reached majority?

Third, as with all longitudinal research, policy and technological changes are likely to affect the execution of biobank research. What, for instance, are the potential implications for reconsent if the definition of “identifiable biospecimen” changes, as is possible under the revised Common Rule? How would such a change be conveyed to currently enrolled participants? Should biobanks put in place more robust consent policies now in anticipation of more stringent requirements in the near future? How can biobanks address the implementation challenges of adopting dynamic consent portals and other systems that serve as a link between biobanks and research participants? While we have focused on regulations within the U.S., different approaches will need to be taken for biobanks outside the U.S. What recontact policies, if any, would be appropriate for pediatric biobanking in other jurisdictions? Would good-faith attempts at recontact be broadly feasible?

Finally, better community engagement could help address the challenges with recontact, especially among racially and ethnically diverse adolescents. Biobanking in the U.S. suffers from a lack of trust among racial and ethnic minority groups (73, 74). How can biobanks build relationships with racially and ethnically diverse adolescents? What are the unique needs and preferences of adolescents from communities with an historical distrust of biobanks? How can biobanks authentically engage with these communities in ways that are respectful to their concerns about participation and still allow them to receive the benefits of biobank-based research?

## Author Contributions

All authors listed have made a substantial, direct and intellectual contribution to the work, and approved it for publication.

## Conflict of Interest

The authors declare that the research was conducted in the absence of any commercial or financial relationships that could be construed as a potential conflict of interest.

## Footnotes

^1^The same study reported that biobanks in the U.S. hold over 200 million specimens total, minors and adults combined. An estimate from a study commissioned by the National Bioethics Advisory Commission suggests that the actual number is now closer to 800 million (8). However, because many of these samples are leftover from clinical tests and de-identified, they were not obtained with consent and consequently would not require reconsent.

^2^45 CFR 46.408 specifies additional conditions for waiving consent that could also apply to reconsent for biobanking but are not as directly relevant as those in section 46.116.

^3^Available from: https://www.hhs.gov/ohrp/regulations-and-policy/guidance/faq/children-research/index.html

^4^Consent forms should still be readable and easy to understand. Consent forms that meet the widely recommended 6th−8th grade reading level can accommodate a range of both adolescents and adults (37).

^5^BioVU. Available from: https://www.vumc.org/dbmi/biovu

^6^Available from: https://www.lebonheur.org/research-and-education/research/biorepository-and-integrative-genomics-initiative/

^7^Available from: https://www.nicklauschildrens.org/medical-services/personalized-medicine-initiative-(pmi)/biobank-and-tissue-repository/for-the-community

^8^Available from: https://biobank.partners.org/

^9^Available from: https://youtu.be/vdJq9IyPhWE

^10^Available from: http://www.geneticalliance.org/programs/biotrust/peer
